# Immune monitoring technology primer: the enzyme-linked immunospot (Elispot) and Fluorospot assay

**DOI:** 10.1186/s40425-015-0074-0

**Published:** 2015-07-21

**Authors:** Sylvia Janetzki

**Affiliations:** ZellNet Consulting, Inc., 555 North Avenue, Ste. 25-S, Fort Lee, NJ 07024 USA

**Keywords:** Elispot, Fluorospot, Immune monitoring, Functional assay

## Description of the technology

Elispot is a workhorse for functional analysis of single immune cells. Originally established for the enumeration of specific antibody-secreting B cells in 1983 [1], the technology has been expanded to the analysis of T cells, NK cells and monocytes. The assay is typically carried out in 96 well plates with a nitrocellulose or PVDF membrane, which is coated with an antibody that is directed against the analyte of interest. Cells and stimulants are added to the plate, and secreted analyte is captured in the immediate surroundings of the cell. After cell removal, bound analyte is made visible via the addition of a biotinylated secondary antibody, avidin-enzyme complex and substrate that precipitates onto the membrane and forms spots which can be automatically enumerated. Each spot represents one cell that secreted the analyte. The most common analytes investigated today are cytokines (IFNɣ, IL-2, IL-5, IL-10, IL-17, Granzyme B, TNF, GM-CSF and many more) and immunoglobulins. Others can also be evaluated, such as chemokines (e.g., CXCL8) or apolipoproteins.

**Fig. 1:**
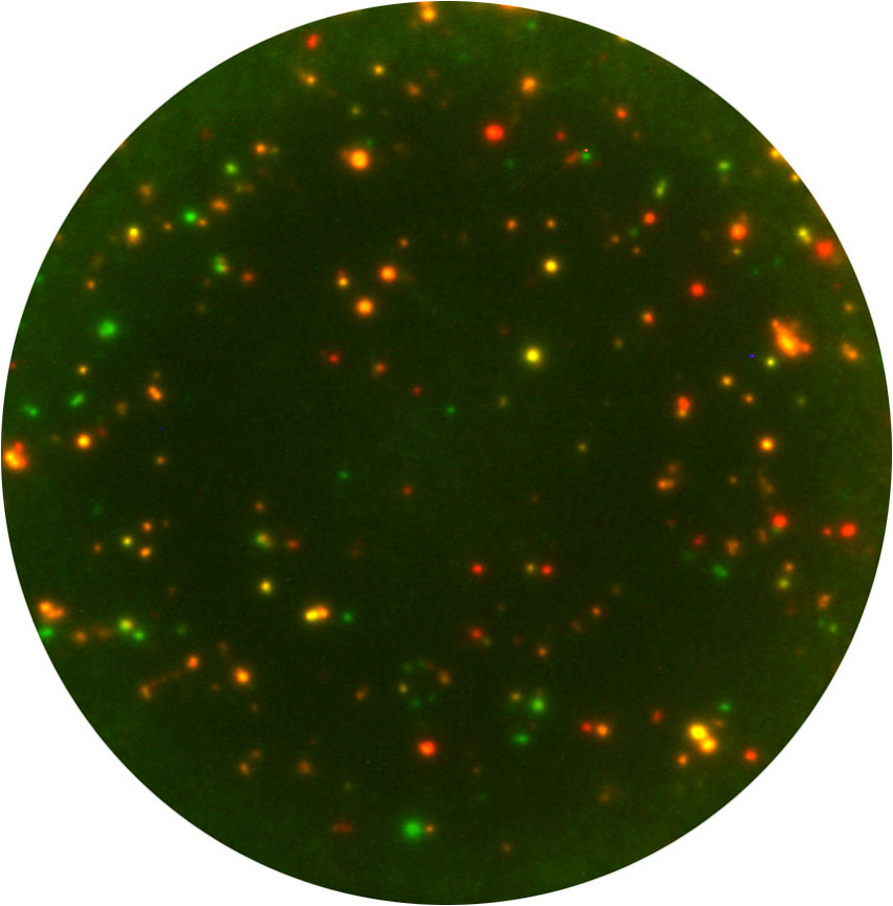
Overlay image of a triple color Fluorospot well. PBMC were tested for the secretion of IFNɣ (FITC spots, green), IL-17A (Cy3, orange), and IL-22 (Cy5, red). The appropriate multi-level evaluation of such sample reveals 7 sub-populations (3 single secretors, 3 dual secretors, 1 triple secretor [11])

The hallmark of the assay is its sensitivity allowing the detection of antigen-specific immune cells in very low frequencies. In contrast to other assays, Elispot measures cells that actually secrete analytes without impairment by receptor binding or protease activity. The assay is easy to perform and transferable, and can be adapted to high throughput. While it was historically limited to the assessment of one parameter per assay, it has now been expanded to the simultaneous assessment of 2 or even 3 parameters in one well by the introduction of fluorescent dyes in the detection cascade (Fluorospot), allowing the identification of up to 7 subsets of immune cells in one well with similar ease of performance as for the enzymatic Elispot evaluating just one parameter (Fig. 1).

Because of its substantial penetration of translational research, Elispot underwent rigorous harmonization efforts led by the cancer immunotherapy field [2], and it is the only functional immune monitoring assay for which a proficiency panel program has been made available for any laboratory independent of affiliation or scientific background (www.proficiencypanel.com).

Lastly, the Elispot assay performance can readily be standardized, qualified, and, if required, validated according to the International Congress of Harmonization (ICH) guidelines [3].

## Type of data obtained/readout

After automated evaluation, spot counts are obtained which represent the number of cells that secreted the analyte of interest per cells plated into a well. Each testing conditioning (or antigenic stimulus) is tested separately, and compared to the negative control counts (cells without antigenic stimulus or with negative control stimulus). The response rate can be determined via empirical rules or statistical testing. Empirical rules present an ad hoc tool with set thresholds based on observations from a study (e.g., more than 10 spots/well and at least 3x above background reactivity). An excellent example on how to establish empirical rules for Elispot has been given elsewhere [4]. For appropriate statistical testing, the variability of Elispot data and the fact that their normal distribution cannot be assumed due to the low number of replicates need to be taken into consideration. Hence non-parametric testing as with the Distribution-free Resampling (DFR) method is recommended [5], for which a free online tool has been made available (http://www.scharp.org/zoe/runDFR/). Further, automated analysis of Elispot plates also provides information on the spot size and spot staining intensity, which may each be correlated with the amount of analyte secreted during the assay time.

## Limitations of the approach

Elispot does not or only to a very limited degree allow the phenotypic analysis of cells that are being assessed. Sub-populations of cells can be isolated prior to the assay in order to identify the responding cell population, what may complicate the antigen presentation requirements for the assay. Blocking with MHC class I or class II antibodies provides another, restricted alternative.

As of today, the functional analysis of immune cells is restricted to a maximum of three parameters, pending the availability of appropriate Fluorospot kits.

## Types of samples needed and special issues pertaining to samples

Single cell suspensions are needed for the assay, such as PBMC or TILs. Cells can be tested directly *ex vivo* without any pretreatment or expansion (even though in vitro expansion may be used for the detection of extremely rare cells). Alternatively, frozen cells can be used after proper thawing and recovery [6]. Similar to the functional assessment of cells by flow cytometry, sample integrity is crucial to the success of Elispot. Whole blood is not suitable for the assay, and needs to be processed for PBMC isolation within a short time frame (typically less than 8 h) in order to prevent the effects of granulocyte activation on T cell functionality.

## Level of evidence

About 5,000 publications exist on the Elispot technique or its use in research, translational or clinical settings. There is a growing body of literature available demonstrating the correlation of the clinical outcome of patients in immunotherapeutic trials with Elispot data [7–9]. Its general clinical validity has further been underlined with the approval of a diagnostic Elispot kit in tuberculosis [10]. The assay is conducted under research as well as GLP/GMP conditions.
